# Nanoparticle-Containing Wound Dressing: Antimicrobial and Healing Effects

**DOI:** 10.3390/gels8060329

**Published:** 2022-05-24

**Authors:** Pavel Yudaev, Yaroslav Mezhuev, Evgeniy Chistyakov

**Affiliations:** Mendeleev University of Chemical Technology of Russia, 125047 Moscow, Russia; yudaevpavel5@gmail.com (P.Y.); valsorja@mail.ru (Y.M.)

**Keywords:** wound, wound dressing, nanoparticle, hydrogel, nanofiber

## Abstract

The dressings containing nanoparticles of metals and metal oxides are promising types of materials for wound repair. In such dressings, biocompatible and nontoxic hydrophilic polymers are used as a matrix. In the present review, we take a look at the anti-microbial effect of the nanoparticle-modified wound dressings against various microorganisms and evaluate their healing action. A detailed analysis of 31 sources published in 2021 and 2022 was performed. Furthermore, a trend for development of modern antibacterial wound-healing nanomaterials was shown as exemplified in publications starting from 2018. The review may be helpful for researchers working in the areas of biotechnology, medicine, epidemiology, material science and other fields aimed at the improvement of the quality of life.

## 1. Introduction

The large surface area and the ultra-fine size, low toxicity and biocompatibility provide the high potential for the use of nanoparticles in the biomedical field [1,2], in dental restorative materials [3,4], in pharmacology as carriers for targeted drug delivery [5,6] including cancer drugs [7]. At the same time, many nanoparticles also show antibacterial action. This has to do with the fact that nanoparticles are much smaller compared with the size of a bacterial cell and can diffuse into the bacterial cell walls with further destruction [8,9,10].

Nanoparticles have a high potential as components of wound dressings being an alternative to antibiotics thanks to the fact that unlike the former, the latter cause fewer side effects and are not prone to causing microbial resistance. This makes it possible to use them for inhibiting the growth of drug-resistant bacteria [11].

It is known that in order to be used in clinical practice the skin regenerating and healing wound dressings must have a range of properties such as biocompatibility, nontoxicity, porosity, as well as air and vapor permeability. They must also sustain a moist environment on the wound surface and absorb exudates, be capable of imitating the extracellular matrix structure and have an antibacterial effect [12]. Such properties can be found in nanofibers and hydrogels based on natural and synthetic polymers modified with nanoparticles. Unlike roller bandage and gauze, hydrogels create a moist wound environment and provide a cooling effect relieving painful sensations in patients [13].

A number of review articles are known dealing with various wound healing materials, i.e., nanofibers modified with silver nanoparticles [14], electroconductive films, membranes, hydrogels [15], nanocomposites based on polylactic acid and zinc oxide nanoparticles [16], polysaccharide antibacterial hydrogels [17,18], hydrogels with copper nanoparticles [19], membranes with gold or silver nanoparticles [20] and others. However, the aforementioned papers do not contain a detailed analysis of antibacterial and healing properties of wound dressings.

The aim of the present paper was to provide an outlook on the use of nanoparticles of metals and metal oxides for production of polymer composite wound dressings through studying their antibacterial and healing action.

## 2. Materials and Methods

The Scopus database was used to conduct the literature survey. The “wound dressing nanoparticles” search returned 1747 results, of which 146 were dated 2022 and 324 were dated 2021, as well as 565 articles from 2018 to 2020. For the purposes of the present analysis 31 most relevant articles published in 2021–2022 were selected, of which 18 articles dedicated to the silver nanoparticles (AgNPs), gold (AuNPs) and copper (CuNPs) nanoparticles. 13 articles dedicated to metal oxides nanoparticles as follows: 7 articles dealing with zinc oxide nanoparticles (ZnO NPs), 2 articles related to iron oxide nanoparticles (FeO NPs or Fe_3_O_4_ NPs), 2 articles on cerium dioxide nanoparticles (CeO_2_ NPs), article dedicated to titanium dioxide nanoparticles with multi-walled carbon nanotubes (MWCNT_TiO_2_), and 1 article on copper oxide nanoparticles (CuO NPs).

## 3. Results

### 3.1. Nanoparticles of Metals

#### 3.1.1. Silver Nanoparticles

The silver nanoparticles obtained by green methods are efficient against pathogens in case of acute, chronic wounds and burns. It is known from the literature [21] that the formation of free radical forms of oxygen is the main mechanism of AgNPs interaction with bacteria leading to damage of the bacteria cell walls.

It is noteworthy that the introduction of AgNPs into the hydrogel matrices is often complicated by low stability of the colloids in aqueous media, which may lead to loss of bioactivity, formation of toxic structure and side effects in patients. Therefore, the biopolymers are normally used for stabilization. For instance, the authors of [22] synthesized AgNPs in the presence of stabilizers in the form of polysaccharides, i.e., ulvan and cellulose. The AgNPs retained its stability even upon loading of a hyaluronic acid-based hydrogel. It was determined that polysaccharides stabilized the AgNPs through the formation of a polyanionic outer layer around the inorganic core attributable to the presence of the hydroxyl, sulphate and carboxyl groups leading to electrostatic repulsion of AgNPs and a high negative zeta-potential value. The addition of polysaccharides did not affect the antibacterial action of AgNPs against *E. coli*, *P. aeruginosa* and *S. aureus*.

**Figure 1:**
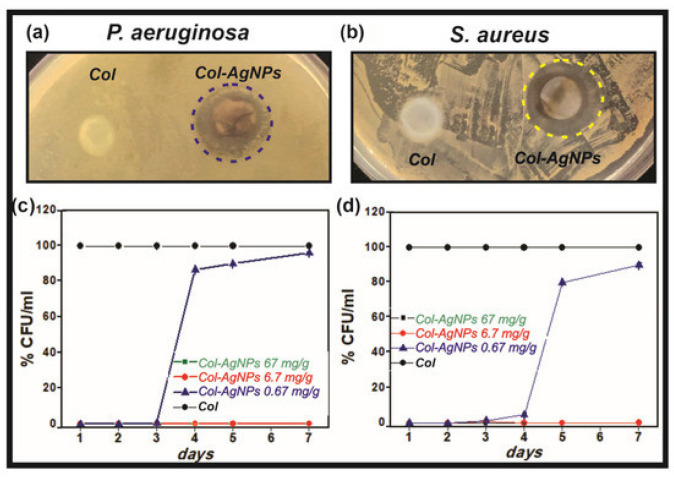
The antimicrobial activity of hydrogels against the *P. aeruginosa* (**a**,**c**) and *S. aureus* (**b**,**d**) bacteria (green and red lines match).

Antezana et al. [23] studied the antibacterial activity and biocompatibility of hydrogels based on collagen and spherical AgNPs ranging from 10 to 15 nm. The growth inhibiting effect of the hydrogels on the *S. aureus* and *P. aeruginosa* was established, as evidenced by the presence of the zones of inhibition vs. the nanoparticle-free hydrogel (Figure 1). The CFU mL^−1^ values for the *S. aureus* and *P. aeruginosa* bacteria significantly decreased within 7 days for the hydrogels containing 6.7 mg g^−1^ and 67 mg g^−1^ of AgNPs. However, for the hydrogels with the lower AgNPs content (0.67 mg g^−1^) the decrease in CFU mL^−1^ was observed only for 3 days followed by a later rise. Unfortunately, the authors did not provide an explanation for this fact.

The viability of the MDCK dog kidney epithelial cells decreased by 80–90% after 48 h of observation for all silver-containing hydrogels. According to the authors this has to do with the release of the toxic silver ions. In order to create biocompatible hydrogels, the *Cannabis sativa* plant extract was used, which provided an antioxidant effect. The MDCK cell proliferation increase by 40–60% was discovered as compared to the extract-free hydrogel. This fact was explained by the authors through the reduction in oxidative stress caused by O_2_, the generation of which is stimulated by AgNPs.

Nešović et al. [24] studied an AgNPs-containing hydrogel with 40–60 nm particles based on polyvinyl alcohol. The germ-kill effect against *S. aureus* (the TL strain) and *E. coli* (the ATCC 25922 strain) was observed within 24 h. In that publication the role of the stabilizing agent was played by polyvinyl alcohol, which prevented the agglomeration and the bioactivity loss of AgNPs. It was determined that the release rate of silver (in the phosphate buffer medium with pH = 7.4) from the hydrogel can be accurately controlled, which is important for clinical use of such wound repair materials and stable protection against infections. According to the authors, the silver-containing hydrogel can be applied for wound healing for a prolonged period of time since it releases the silver slowly and maintains the required concentration thereof for 28 days. However, the authors of paper [24] did not study the mechanical properties of the hydrogel.

Santiago-Castillo et al. [25] the antibacterial properties of nanofibers based on high molecular weight polyvinyl alcohol (146–186 kDa), chitosan (190–310 kDa) and cube-shaped AgNPs were investigated. A combination of chitosan with polyvinyl alcohol was used since the solutions of chitosan are highly viscous at room temperature due to the polycationic nature of chitosan in solutions and due to the rigid structure, which complicated the production of fiber by means of electrospinning. The nanofibers demonstrated antibacterial activity against the *E. coli* (the ATCC 25922 strain) and the *S. aureus* bacteria (the ATCC 25923 strain). The diameters of the zones of inhibition were equal to 22 mm and 20 mm for *E. coli* and *S. aureus*, respectively. The antibacterial activity of the obtained nanofibers was better compared to the nanofibers containing ZnO NPs and copper nanoparticles. The hardness increase in the silver-containing fiber from 32 hPa to 152 hPa compared with the polyvinyl alcohol-chitosan fiber was established.

In contrast to Santiago-Castillo et al. [25], the authors of [26] used castor oil for loading of the composite instead of chitosan in order to increase the antibacterial properties of the polyvinyl alcohol and AgNPs hydrogels. The silver nanoparticles at that were synthesized in the presence of the *Mentha piperita* leaf extract and then dispersed in the polymer matrix. The nanoparticles were equidistributed in the polymer matrix without aggregation. The films were obtained by green method using environmentally friendly raw materials, water and ethanol as solvents. The formulated films displayed the growth inhibition zone diameters of 8.33 mm and 9 mm for the *S. aureus* and *P. aeruginosa* bacteria, respectively. At the same time, the obtained values were lower than in [25] for *S. aureus* and lower than that of commercially available antibiotics amoxicillin (13.7 for *S. aureus*) and amikacin (19.7 for *P. aeruginosa*).

A burn-repair hydrogel was developed, in which oxidized dextran, adipic dihydrazide grafted hyaluronic acid and quaternized chitosan were used as the polymer matrix and the spherical 50–100 nm AgNPs was used as the filler [27]. The reduction in the silver ions Ag^+^ to AgNPs was performed by placing the gel into 0.1 M aqueous solution of silver nitrate. The hydrogel showed antibacterial effect against *E. coli*, *S. aureus* and *P. aeruginosa*. The diameters of the zones of inhibition growth for *E. coli*, *S. aureus* and *P. aeruginosa* were 16, 20 and 17 mm, respectively. The in vivo study of the healing effect showed a decrease in the burn area in the SD male rats after 7 days of observation. The wound was completely healed after 14 days of observation, whereas in the control group (AgNPs-free gel) the healing occurred only on the 21st day. Furthermore, the histological examination showed full re-epithelization and a pronounced deposition of collagen after 21 days of observation and the immunohistochemistry assay demonstrated the expression reduction in the inflammatory cytokines IL-6, IL-1β and TNF-α on the 14th day.

Since the obtained hydrogel had a pronounced antibacterial, healing and anti-inflammatory effect it can be used for fast healing of burns. However, the study of cytotoxicity on the L929 mice fibroblast cells showed less than 70% cell viability, which is indicative of the toxicity of the silver-containing hydrogel.

The authors of paper [28] produced the antibacterial nontoxic hybrid nanofibers based on quaternized chitin, tannic acid, polylactic acid, and polyurethane with the addition of AgNPs using the electrospinning method. The obtained nanofibers inhibited the growth of the Gram-positive *S. aureus* bacteria and the Gram-negative *E. coli*. Noteworthy, the authors chose to express the diameter of the inhibition zone in nm and not in mm, which does not match the indications on the Petri dishes. This does not allow for a high-quality data interpretation.

Bozkaya et al. [29] studied the antibacterial and wound-healing effects of the AgNPs- containing (particle size 14.8 nm) polycaprolactone and polyethylene oxide fibers. The water-methanol extract of the *Centella asiatica* plant was used in the role of the stabilizer and the reductive agent. The fibers had the sufficient mechanical properties (tensile strength min. 2.5 MPa) for wound-dressing applications, porosity (pore size min 1000 mm), water absorption, water vapor transfer rate (in the range of 2000–2500 g m^−2^ day^−1^), air permeability, antibacterial effect (against *S. aureus*, *E. coli*, *C. Albicans*) and biocompatibility. The diameters of the zones of inhibition for the resulting fibers grew along with the rise of the AgNPs concentration. The inhibition zone diameters after 24 h of incubation were 24, 21 and 21 mm for *S. aureus*, *E. coli*, and *C. albicans*, respectively. The L929 cell viability was above 85%, which indicates that the fibers are nontoxic. The authors plan to use the obtained nanomaterial for healing of burns. That being said, the in vivo studies of the fibers’ healing and anti-inflammatory effects are needed for their introduction into clinical practice.

The authors of [30] manufactured a high-swelling (4000%) cryogel based on gelatin and AgNPs (particle size 10–20 nm). The cryogel was active against the methicillin-resistant *S. aureus* (MRSA) and *P. aeruginosa* and promoted regeneration of the burn wound tissue in the Kunming female mice weighing 30–35 g. The wound area decreased after 2 weeks. The wound contraction was 96%, whereas that of the commercially available Tegaderm wound dressing was only 56%. The expression of the TNF-α cytokines was decreased compared to Tegaderm within 1 week of treatment, which indicates better anti-inflammatory properties of the cryogel. The cryogel also possessed the ability to absorb blood and showed haemostatic action since it was able to decrease the blood loss from 350 mg down to 81 mg. The authors proposed the use of the cryogel for the production of burn wound dressings.

**Figure 2:**
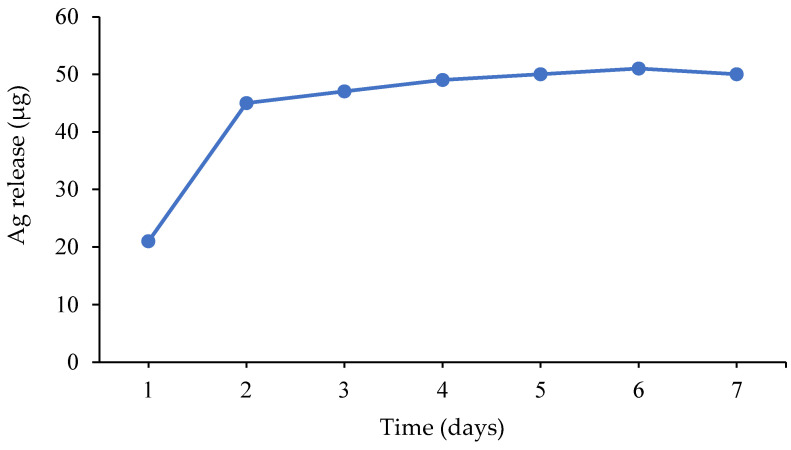
The release of silver from the hybrid hydrogel in vitro.

Since gelatin yields high-swelling gels it can increase the same parameter for other polymers as well. Thus, Sethi et al. [31] synthesized a hybrid hydrogel containing spherical (particle size 4–19 nm) and quasispherical (particle size 4–58 nm) AgNPs based on starch and gelatin. N,N’–methylenebisacrylamide was used for chemical crosslinking of the polymers, whereas for physical crosslinking polyacrylic acid was grafted to starch and gelatin. The maximum swelling degree in blood, water, 0.9% aqueous solutions of MgCl_2_ and NaCl for the hybrid gel were by 12–27% higher compared to that of the starch-based gel and the compressive modulus grew by 201%. The hybrid hydrogel possessed sufficient porosity, antibacterial activity against *E. coli* and *S. aureus* and was non-cytotoxic towards the human skin fibroblasts (cell viability 89%). The release of silver into the phosphate-buffered solution was fast within 2 days, whereas later it became nearly stable (Figure 2). The amount of the released silver varied in the range of 21–51 mcg per 0.5 g of gel, which is acceptable for use in the wound-repair application.

It is often the case that starch requires other types of modification. Due to low solubility in organic solvents, it is difficult to obtain starch-based nanofibers by the electrospinning method. For that reason, the starch is chemically transformed into hydroxypropyl starch and for mechanical strengthening of the nanofibers it is blended with synthetic polymers. For instance, El-Hefnawy et al. [32] obtained nanofibers for wound dressings on the basis of hydroxypropyl starch, polyurethane, and AgNPs with particle size below 5 nm. The AgNPs was synthesized by green method in the presence of the *Nerium oleander* leaf extract as the stabilizer and the reductive agent. Four polymer compositions were prepared for electrospinning containing 0 (AgNPs-0@NFs), 1 (AgNPs-1@NFs), 2 (AgNPs-2@NFs) and 3 (AgNPs-3@NFs) ml of AgNPs water dispersion. The full composition of the formulations is shown in Table 1.

The obtained nanofibers showed an antibacterial effect against wound-present pathogens including the drug-resistant Gram-negative *P. aeruginosa* bacteria. The zones of inhibition diameters values (ZOI) are presented in Table 2. It was determined that the highest ZOI values can be observed for the AgNPs-3@NFs nanofibers, whereas the lowest were seen in the case of AgNPs-1@NFs. Furthermore, 2 the ZOI values for AgNPs-3@NFs were much higher than that of the commercially available ciprofloxacin antibiotic.

A composite based on the cellulose nanofibers and AgNPs was developed and studied in terms of its antimicrobial properties [33]. The zone of inhibition diameters for *E. coli*, *P. aeruginosa*, *S. aureus*, *B. subtilis*, *P. mirabilis* and *C. albicans* was 8.7, 8.0, 10.7, 11.0, 10.7 and 10.3 mm, respectively. The cellulose nanofibers prevented the aggregation of AgNPs and the loss of their bioactivity. The silver ions Ag^+^ were adsorbed on the negatively charged surface of the nanofibers, and thanks to the carbonyl groups on the surface thereof, were reduced to silver nanoparticles. The obtained composite was more active against the abovementioned microorganisms compared with the commercially available gentamycin antibiotic. It is planned to use the composite as a dressing for repairing wounds of various origin.

Rao et al. [34] obtained a gel based on carboxymethyl chitosan and AgNPs stabilized by tannic acid. The silver nanoparticles were of spherical shape 5 nm in size. The hydrogel displayed antibacterial action against *E. coli* and *S. aureus*, good adhesion and cell proliferation; it was nontoxic to the CCDK skin fibroblast cells. The diameters of the zones of growth inhibition for *E. coli* and *S. aureus* were 19.2 mm and 17.5 mm, respectively. Unfortunately, the publication [34] does not contain a study of the gel’s mechanical properties, whereas the gel must be sufficiently strong to withstand the external forces during motion of the human body.

Yan et al. [35] studied the mechanical properties of the hydrogel membrane on the basis of AgNPs-containing calcium alginate-polydopamine-carboxymethyl chitosan. The membrane itself was prepared by immersion of the calcium alginate-polydopamine-carboxymethyl chitosan film into a silver nitrate water solution. It was determined that the tensile strength and the elongation at break were by 342.99% and 13.84% higher vs. the calcium alginate membrane and were equal to 91.92 MPa and 2.55%, respectively. It was also reported that the hydrogel membrane showed antibacterial activity against *E. coli* and *S. aureus*. The diameters of the growth inhibition zones for the *E. coli* and *S. aureus* bacteria were 14 mm and 13 mm at the silver nitrate concentration 2 mM. The membrane was nontoxic at the silver nitrate concentration of 2 mM since the viability of the HSF human skin fibroblast cells was over 70%. However, as the silver nitrate concentration increased from 2 mM to 4 mM the cell viability dropped down to 10%, which is indicative of the membrane’s cytotoxicity. This means that in order for the membrane to be applied as a wound dressing the AgNO_3_ concentrations must be low.

The polyacrylonitrile nanofibers containing curcumin, tannic acid and AgNPs (average particle size 19.1 nm) are active against *E. coli* and *S. aureus* and are biocompatible with the MSCs mesenchymatous stem cells [36]. The tannic acid played the role of the reducing agent for the silver ions in the production of AgNPs.

The maximum diameters of the growth inhibition zones for the *E. coli* and *S. aureus* bacteria were 1.1 and 1.2 cm. It was also established that the silver is released from the fibers for a prolonged period of time (more than 6 days).

In view of this, the AgNPs are a promising component for the wound dressings. The materials based on polymers and AgNPs have an antibacterial, antifungal, healing and anti-inflammatory effect and demonstrate a lasting prolonged release of silver from the polymer matrix [29,31]. The high-water retention capacity of the materials containing AgNPs ensures the absorption of the wound exudates. It is attributable to the hydrogen bonds and the dipole–dipole interactions between the polar (-OH, -COOH) groups within the AgNPs structure stabilized by the plant extracts and water [29].

Noteworthy, the silver nanoparticles applied to the skin in the dispersion form did not cause aggressive skin rashes in rabbits and swine [37]. However, the AgNPs may come into contact with keratinocytes upon the skin barrier damage, e.g., in case of burns and chronic wounds. In publication [38] the toxic effect of AgNPs towards the human skin keratinocytes (the HaCaT cell line) was reported, i.e., the main epidermal cells. Additional in vitro and in vivo studies of AgNPs are needed in order to avoid side effects for application in wound dressings.

#### 3.1.2. Nanoparticles of Gold

Zhang et al. [39] developed a nanoporous hydrogel dressing based on heparin and polyvinyl alcohol containing spherical AuNPs with the particle size below 100 nm. The dressing inhibited the growth of the *S. aureus* and *E. coli* bacteria, improved healing of wounds in the Kunming mice; it was nontoxic towards the HFFF2 human dermal fibroblasts (cell viability over 90%). It was suggested to use the dressing for burn repair. The spherical gold nanoparticles of various sizes are nontoxic to human skin [40]. However, the AuNPs-containing polycaprolactone nanofibers showed a minor antimicrobial effect against the *S. aureus*, *E. coli*, *P. aeruginosa*, *C. albicans* (the zone of inhibition values below 3 mm) [41].

#### 3.1.3. Nanoparticles of Copper

In paper [42], a double-layer nanofiber was synthesized. The first layer was made of polyvinyl alcohol and chitosan containing CuNPs, whereas the second layer consisted of poly-N-vinyl pyrrolidone. The high antibacterial activity against the Gram-positive *S. aureus* (15.6 ± 1.1 mm) and *B. cereus* (29.6 ± 0.42 mm) and the Gram-negative *E. coli* (13.3 ± 0.8 mm) and *P. aeruginosa* (10 ± 1 mm) bacteria was established. The authors also showed the faster healing in the albino Wistar rats on the 3rd day of observation vs. the controls (nanoparticles-free gel). On the 16th day, the wounds healed completely. The nontoxicity of the CuNPs as compared to the AgNPs towards the human skin keratinocytes is advantageous [43]. However, the activity of the copper-containing nanofiber against the *S. aureus*, *E. coli* and *P. aeruginosa* microorganisms was lower even than that of the tetracycline antibiotic [42].

### 3.2. Nanoparticles of Metal Oxides

#### 3.2.1. Wound Dressings Containing Zinc Oxide Nanoparticles

ZnO NPs inhibit the growth of wound microbial population, improve healing, promote tissue regeneration and wound contraction. Bandeira et al. [44] synthesized nanofibers based on polyacrylic acid and polyallylamine hydrochloride modified with spherical ZnO NPs 18 ± 5 nm in diameter by means of electrospinning. The nanoparticles were synthesized by green method using the *Ilex paraguariensis* leaf extract. The ZnO NPs content in the fiber was 11.2 wt.%. The nanofibers showed the morphology similar to that of the skin extracellular matrix and inhibited the growth of both Gram-negative *E. coli* (the ATCC 35218 strain) and the Gram-positive *S. aureus* (the ATCC 25923 strain). The nanofibers decreased the viability of the *S. aureus* and *E. coli* bacterial cells by 65% and 10%, respectively. Thus, the Gram-negative *E. coli* are more resistant to the zinc oxide nanoparticles as compared to the *S. aureus* bacteria.

The paper [45] demonstrated that the hydrocolloid patch with ZnO NPs from CGBio (Seoul, South Korea) improves wound healing in the Sprague Dawley rats weighing 200–300 g. A decrease in the wound area was observed in comparison to the control group after 10 days. The wound healed by 98% on the 10th day vs. day 0. The microscopic study showed a thicker outer skin and granulation tissue layers as well as higher collagen on the 10th day compared to the control. The IF staining showed a decrease in the CD68 anti-inflammatory cytokines concentration by ca. 30%, the IL-8 by 50%, the TNF-α by 50%, the MCP-1 by 50%, the IL-6 by 90% and the IL-1β by 20% in the 10th day. Moreover, higher levels of the α-CMA, TGF-β3 fibroblasts biomarkers, as well as those of vimentin and M2 were determined vs. controls, which is critical during the wound healing process. Therefore, the obtained patch had the necessary healing and anti-inflammatory effects. Furthermore, the authors plan to investigate the cytotoxicity of the produced material. However, the authors of [45] did not name the polymer from which the hydrocolloid patch was made and what materials and solvents were used for its preparation, which is not entirely meaningful from a scientific standpoint.

In publications [46,47] biocomposite chitosan-based films modified with ZnO NPs were prepared that were active against wound-present pathogens, i.e., *E. coli*, *S. aureus*, *K. pneumoniae*, *B. subtilis*. The work [46] dealt with the analysis of a film based on chitosan (mol. weight 800 kDa), glycyrrhizinic acid and ZnO NPs/palygorskite nanorods. The film had a high degree of swelling of 472.49%, which indicates the fact that the film can easily absorb the wound exudates. The agar diffusion method was used for qualitative evaluation of the antibacterial effect of the films against the pathogens such as *E. coli* and *S. aureus* as well as the drug-resistant β-lactamase-producing *E. coli* (ESBL-*E. coli*) and MRSA; for the quantitative evaluation the colony count method was used. At 5 wt.% content of nanorods the film inhibited 99.5% *E. coli*, 99.8% *S. aureus*, 99.6% ESBL-*E. coli* and 99.8% MRSA. The diameters of the growth inhibition zones for *E. coli*, *S. aureus*, ESBL-*E. coli* and MRSA were 15.04 ± 1.07 mm, 14.50 ± 0.42 mm, 13.28 ± 0.31 mm and 13.64 ± 0.29 mm, respectively. The ZnO NPs release the zinc ions, which bind to the negatively charged of the bacterial cell membrane causing lysis. It is necessary to conduct an in vivo study of the wound surface evolution over time.

In manuscript [48], the authors developed a wound dressing on the basis of cotton pads and a biocomposite consisting of chitosan, glycogen and 30–80 nm ZnO NPs. Contrary to publications [46,47], in [48] the healing effect of the material was studied as compared to sterile gauze. A fast and almost complete healing of back wounds in the Wistar rats was demonstrated (males weighing 180–200 g), as well as excellent epithelization, granulation, tissue generation and collagen deposition. In 3 days, the wound diameter decreased by 11.7%, whereas the wound area decreased by 22.5%, which is more than double of that in the control group (sterile gauze). After 17 days of observation, the size of the wounds decreased by 99.7% vs. 89% in the control group. Additionally, the dressing had a significant antibacterial effect against the wound bacteria *P. aeruginosa*, *S. aureus*, *S. epidermidis* and a fungicidal effect against the causal fungus *C. albicans*. The decrease percentage of the CFU for the Gram-negative *P. aeruginosa* bacteria and the *C. albicans* fungus was lower than that for the Gram-positive bacteria *S. epidermidis* and *S. aureus* and was equal to 35%, 63%, 97% and 85%, respectively. The antibacterial action was explained by the authors through the electrostatic attraction between the positively charged dressing and the negatively charged bacterial cell walls. According to the authors, the prepared wound dressing can be used in clinical practice for treatment of chronic wounds and diabetic foot sores. However, the paper did not study the material’s cytotoxicity and other important parameters applicable to wound dressings.

The antibacterial and healing effects of the hydrogel of another mixed polymer composition, i.e., polyvinyl alcohol, chitosan and starch with addition of ZnO NPs (particle size below 30 nm) was studied [49]. *E. coli* (the ATCC 25922 strain) and *S. aureus* (the ATCC 25923 strain) were selected as the test microorganisms. The healing effect was studied in vivo on the male Wistar rats (200–250 g). The minimum inhibiting concentration values against *E. coli* and *S. aureus* were 200 μg mL^−1^ and 50 μg mL^−1^, respectively. On the 7th day of the in vivo study the wound contraction percentage was lower than in the control group, whereas 100% wound contraction was observed on the 14th day. The microscopic wound repair investigation showed 60% collagen fiber formation after 7 and 14 days, which is necessary for the regeneration of blood vessels, whereas in the control group it was under 50%. It was established that the viability of the L-929 and HDF cells was over 75%, which suggests the nontoxicity of the dressing. Additionally, the hydrogel had a porous structure (pore size 20 ± 8 µm), the optimal degree of swelling (swelling ratio 5.5–6.7) and the water vapor transmission rate (in the range of 2000–3000 g m^−2^ day^−1^). Furthermore, the addition of ZnO NPs improved the hydrogel’s tensile strength. The formulated material had great prospects in terms of the wound dressing production.

With that said, the wound dressings containing the ZnO NPs are a promising type of materials for chronic wound repair and diabetic foot sores treatment thanks to their improved healing effect and antimicrobial action compared to cotton gauze. However, high concentrations of ZnO NPs (50 μg mL^−1^ and above) did inhibit the proliferation of the HGF-1 human gingival fibroblast cells [50].

It should be noted that along with all of its positive properties, the ZnO NPs are potentially genotoxic towards the human epidermal cells even at low concentrations (below 1 μg mL^−1^) [51]. In the review paper [52] an analysis of the liver toxicity, lung toxicity, neurotoxicity and immunotoxicity studies of the ZnO NPs was performed. It was established that the ZnO NPs toxicity depends on the concentration/dose, administration route, exposure time and the particle size. This means that further toxicity study of the ZnO NPs is required, and they should be applied with caution.

#### 3.2.2. Wound Dressings Containing Nanoparticles of Iron Oxides

In publication [53], a porous nanocomposites based on chitosan, polyvinyl alcohol and the FeO iron oxide nanoparticles (particle size under 50 nm) was obtained. The nanoparticles were prepared using the *Pinus densiflora* leaf extract. The nanocomposite containing 0.01 wt.% of nanoparticles had a large zone of bacterial growth inhibition with respect to *B. cereus* (22 mm), *S. aureus* (21 mm), *E. coli* (20 mm), *S. enterica* (22 mm). The inclusion of the nanoparticles into the polymer matrix increased proliferation of the HEK923 cells (the cell line derived from human embryonic cells) in comparison with chitosan, which improved the healing of wounds. It was suggested by the authors to use the obtained composite for diabetic sores repair. However, a further in vivo study is needed.

Paydayesh et al. [54] synthesized a polyhydroxyethyl methacrylate hydrogel with addition of the 20–40 nm Fe_3_O_4_ iron oxide nanoparticles. The swelling index of the gel decreased along with the nanoparticles content growth, which has to do with the nanoparticles acting as crosslinking centers. The gel containing 15 wt.% iron oxide nanoparticles inhibited the growth of the *E. coli* and *S. aureus* bacteria. Additionally, the obtained nanocomposite hydrogel was nontoxic towards the HFFF2 fibroblast cells, which makes its application in the wounds dressing area possible. However, the Fe_3_O_4_ iron oxide nanoparticles decreased the viability of the human keratinocytes at concentrations exceeding 50 μg mL^−1^ [55].

#### 3.2.3. Wound Dressings Containing Cerium Dioxide Nanoparticles

Zamani et al. [56] established that the gelatin-polycaprolactone nanofibers containing spherical cerium dioxide nanoparticles (max. 20 m) show an antibacterial effect against *P. aeruginosa*. The minimum bactericide concentration was equal to 50 μg mL^−1^. It was also reported that the expression of the *shv*, *kpc*, *imp* genes found in the resistant *P. aeruginosa* strains was decreased in the presence of the nanofibers. The nanofiber containing 200 μg mL^−1^ of CeO_2_ NPs was nontoxic since 97% of the human fibroblast cells (cell line HU 2) survived. The developed fiber was proposed for use in production of dressings for skin infection treatment.

The CeO_2_ cerium dioxide nanoparticles were used for preparation of the nanofiber on the basis of poly-L-lactic acid and gelatin [57]. The fibers demonstrated no cytotoxicity towards the NIH 3T3 mice fibroblast cells. The healing effect of the membrane was evaluated via a model back skin burn on the SD rats weighing 200 g. On the 10th day of observation, the open area decreased considerably. On the 21st day the wounds healed contrary to the control group. The scars were minimal, and the healing effect was better compared to the control (medical patch). It was suggested to use the developed fibers as a cheap material for wound dressings. This being said, the antimicrobial effect of the nanofiber was not studied in the present paper. Respective additional research is required for application of the material in the medical field.

The cerium oxide nanoparticles showed a low toxicity towards the HaCaT human skin keratinocytes [58]. However, the provided toxicological information is insufficient and the genotoxicity and the apoptosis studies for the CeO_2_ NPs are required.

#### 3.2.4. Wound Dressings Containing Titanium Dioxide Nanoparticles

**Figure 3:**
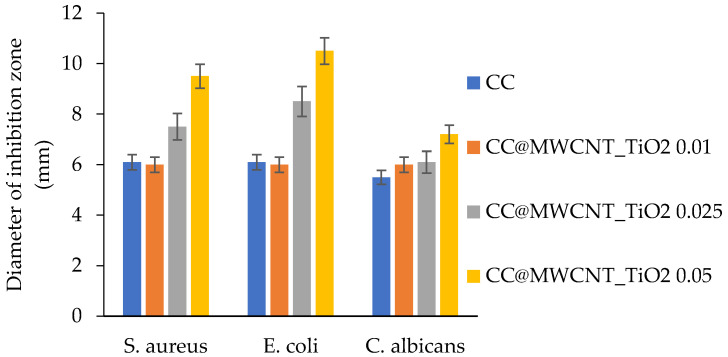
The antimicrobial activity of the films against the *S. aureus*, *E. coli* bacteria and the *C. Albicans* fungi. CC—cellulose acetate-collagen, MWCNT—multi-walled carbon nanotubes, 0.01, 0.025, 0.05—the weight (g) of MWCNT_TiO_2_ added to the solution of cellulose acetate and collagen in acetic acid and water.

David et al. [59] manufactured a nanocomposite on the basis of multi-walled carbon nanotubes with spherical titanium dioxide nanoparticles (ca. 15 nm in diameter) on their surface (MWCNT_TiO_2_). The nanocomposite was loaded into a cellulose acetate-collagen porous film. The antimicrobial action, biocompatibility and cytotoxicity of the obtained films were investigated. By means of a diffusion test it was determined that the films inhibit the growth of the *S. aureus* and *E. coli* bacteria, as well as the *C. Albicans* fungi. The largest inhibition zone was observed for the Gram-negative *E. coli* bacteria (Figure 3).

The diameters of the inhibition zones grew along with the growth of the MWCNT_TiO_2_ content in the film. Fluorescent microscopy showed that the obtained films were nontoxic towards the HDFn human dermal fibroblast cells, which participate in the wound healing process. However, the paper [59] does not contain any in vivo studies of the obtained films.

It should be noted that the titanium dioxide nanoparticles of various sizes (10 nm, 21 nm, 32 nm) did not show any statistically significant effect on the viability of the HaCaT human keratinocyte cell line [60,61].

#### 3.2.5. Wound Dressings Containing Copper Oxide Nanoparticles

In publication [62], the antibacterial properties of the nanofibers based on polycaprolactone and gelatin containing 1 wt.% CuO NPs were studied. It was determined that the nanofibers have a strong antibacterial activity against the Gram-positive *S. aureus* (51 ± 1.2 mm), the multidrug resistant *S. aureus* (40 ± 1.7 mm) and the Gram-negative *P. aeruginosa* (31 ± 0.5 mm) and the *E. coli* (30.5 ± 0.3 mm) bacteria, which was explained by the authors through generation of the reactive oxygen intermediates that destroy bacterial cells by cell membrane oxidation. At that, the CuO NPs may be toxic to NIH3T3 [63].

Moreover, it was established that the CuO NPs were toxic towards the HaCaT cells at concentrations exceeding 5 μg mL^−1^ after 24 h of exposure as they damage the cell membranes and are genotoxic [64], which limits their use as part of wound dressings.

## 4. Discussion

From the reviewed publications, it may be concluded that both natural (hyaluronic acid, chitosan, carboxymethyl chitosan, cellulose, cellulose acetate, collagen, gelatin, starch, hydroxypropyl starch, sodium alginate) and synthetic polymers (polyvinyl alcohol, polycaprolactone, polyethylene glycol, polyacrylonitrile, polyurethane, polylactic acid, polyhydroxy ethyl methacrylate, and polyacrylic acid) or blends thereof are used for the production of modern wound-repair materials. The polymers provide biocompatibility, swelling, porosity, as well as favorable conditions for tissue regeneration. However, the antimicrobial action of these polymers is oftentimes insufficient. Therefore, nanoscale fillers are additionally introduced into the polymer matrix that provide the antibacterial effect, such as silver, gold, copper, zinc oxide, cerium dioxide, iron oxides (FeO, Fe_3_O_4_) and titanium dioxide nanoparticles with multi-walled carbon nanotubes. Table 3 shows the characteristics studied for these composites.

The antimicrobial activity was studied in most of the reviewed publications (25 paper) as well as cytotoxicity and biocompatibility (16 papers), and mechanical properties (13 papers) of the materials. However, the in vivo wound healing activity studies of the materials can be found only in 7 articles. Additionally, only 5 publications were dedicated to the water vapor transmission rate, the water absorption and air permeability studies that are necessary for the optimal balance of moisture and oxygen in the wound. More systematic studies of the materials in terms of their healing effect need to be carried out on large animal models (e.g., horses), as well as air and vapor permeability and water absorption must be investigated in order to evaluate the medical application potential thereof.

When applying nanoparticles in the wound-repair products, their activity and the antimicrobial action should be considered since the particles may also affect various tissue cells in patients leading to all kinds of complications (Table 4).

An additional survey of the literature published on the subject starting from 2018 was conducted in order to evaluate the application trends for the nanoparticles as antimicrobial preparations in wound dressings. The results are presented in Table 5.

From the results presented in Table 4, it can be concluded that the largest share of the research is dedicated to the preparation of the dressings containing silver nanoparticles, i.e., 37 papers. At the same time, the distribution of the research over a five-year period is not always uniform. Nevertheless, in recent years the number of publications on wound-repair AgNPs-based nanomaterials had increased. The materials based on zinc oxide nanoparticles are relatively actively studied (18 articles). Other nanoparticles are much less widely used, which, based on the results, has to do with the weaker antimicrobial action vs. AgNPs and ZnO NPs. Furthermore, the AgNPs and ZnO NPs are less toxic at low concentrations towards the live mammalian cells. At that the silver nanoparticles are more promising in terms of the practical application than ZnO NPs since the latter are potentially genotoxic at elevated concentrations. It must be taken into account that in order to achieve the antimicrobial effect while not harming the patient at the same time is hardly possible by only varying merely the concentration of the nanoparticles. Therefore, the evaluation of the trends in the field of application of polymers for antibacterial wound dressing matrices is equally important because the polymer defines many characteristics of the obtained nanomaterial. The literature survey results are presented in Table 6.

As it can be seen from the table, among the polymer matrices for wound-repair nanomaterials over the past 5 years the most publications were dedicated to biopolymers (66 papers), whereas 44 papers were dedicated to synthetic polymers. The polysaccharides were leading among the biopolymers with the number of publications reaching 50. Chitosan also had a stable leading position. The number of studies dedicated to the use of cellulose had decreased in recent years, whereas that of starch had on the contrary increased. In 2021–2022, some publications emerged dealing with wound dressings based on heparin, glycogen and quaternized chitin. Gelatin is another example of a biopolymer with growing interest.

Polyvinyl alcohol is the synthetic polymer most frequently used in wound-repair nanomaterials. It was mentioned in 19 articles over the abovementioned time period, whereas the distribution from one year to another varied insignificantly. Polycaprolactone was used in the studies to a lesser extent but with similar consistency. The newest articles published in 2022 are presenting the research dedicated to polymers such as polyacrylonitrile, polyhydroxyethyl methacrylate and polylactic acid, among which the latter being the most promising.

## 5. Conclusions

Of note, the hydrogels, fibers and membranes containing silver nanoparticles have been studied the most in terms of their antibacterial, fungicidal and cytotoxic properties. The AgNPs have a pronounced antibacterial action and often surpass certain antibiotics with respect to their efficiency against the gram-positive *S. aureus* and the gram-negative *P. aeruginosa* bacteria being the main pathogens present in wounds. This makes it possible to use AgNPs in the composition of wound dressings, mainly for burn repair. However, the silver nanoparticles are prone to aggregation, which may lead the change of scale and the loss of antimicrobial action. For this reason, the development of materials in which the silver is uniformly distributed in the polymer matrix is a hot topic for further research [105].

As far as the wound dressing matrices are concerned, despite a wide range of polymers analyzed in many studies, only two have the potential for practical application, i.e., chitosan, the biopolymer, and the synthetic polyvinyl alcohol.

The low molecular weight chitosan is nontoxic; it has healing, haemostatic, pain-relieving and antimicrobial effects [106]. The polymer is rather easy to obtain while the raw material for its production is abundant in nature including food production by-products. Additionally, no skin reactions to chitosan dressings in patients allergic to mollusks were reported [107]. However, there were no studies of potential side effects and allergic reactions to the polymer materials in the reviewed publications, such as allergic contact dermatitis or toxicodermatosis.

The polyvinyl alcohol is also relatively cheap, easy to produce, nontoxic and allows for obtaining a material with a preset molecular weight; it does not contain any toxic monomer admixtures and solvents, which may be found in other polymers. What is especially important, polyvinyl alcohol is easy to modify, which allows regulating the parameters of a PVA-based gel and grafting various functional groups, substances and preparations to its polymer chain. Thanks to modification, this polymer can provide a wide range of properties and it can be used under various conditions.

Thus, the antimicrobial wound-repair materials based on polyvinyl alcohol or chitosan modified with silver nanoparticles have the broadest practical application potential.

## Figures and Tables

**Table 1:** The lists of the utilized volumes of polymers and AgNPs used in the preparation of electrospinning solutions (PU—polyurethane (12 wt.% solution in DMF), HPS—hydroxypropyl starch (15 wt.% solution in DMSO)).

Composition Code	PU Volume (mL)	HPS Volume (mL)	Water Dispersion AgNPs (mL)	Total Volume (mL)
AgNPs-0@NFs	10	5	0	15
AgNPs-1@NFs	9	5	1	15
AgNPs-2@NFs	8	5	2	15
AgNPs-3@NFs	7	5	3	15

**Table 2:** The antimicrobial activity and ZOI diameters for AgNPs-0@NFs, AgNPs-1@NFs, AgNps-2@NFs, and AgNPs-3@NFs against human-associated pathogens.

Composition Code	ZOI Diameters (mm)			
	*P. aeruginosa*	*E. faecalis*	*C. albicans*	*A. niger*
AgNPs-0@NFs	0 ± 0	0 ± 0	0 ± 0	0 ± 0
AgNPs-1@NFs	15 ± 0.20	13 ± 0.18	10 ± 0.16	11 ± 0.23
AgNPs-2@NFs	21 ± 0.17	19 ± 0.12	17 ± 0.20	15 ± 0.15
AgNPs-3@NFs	26 ± 0.23	24 ± 0.25	23 ± 0.23	21 ± 0.17
Ciprofloxacin	12 ± 0.19	11 ± 0.25	8 ± 0.21	7 ± 0.14

**Table 3:** The characteristics of the wound dressings studied in the presented publications.

Nanofiller	The Studied Characteristics of the Wound Dressings	Ref.
AgNPs	Antimicrobial activity	[22,23,24,25,26,27,28,29,30,31,32,33,34,35,36]
	Cytotoxicity analysis	[22,27,29,31,34,35]
	Biocompatibility	[22,28,30]
	Silver release measurements	[24,29,31,36]
	In vivo wound healing activity	[27,30]
	Swelling degree	[27,30,31]
	Porosity	[29,31]
	Mechanical properties	[25,28,29,31,35,36]
	Immunohistochemical analysis	[27,30]
	Water absorption	[29]
	Water vapor transmission rate	
	Air permeability	
	Hemocompatibility	[30,31]
	In vitro cell compatibility	[36]
AuNPs	Antimicrobial activity	[39,41]
	Cytotoxicity analysis	[41]
	Biocompatibility	[39]
	In vivo wound healing activity	[39]
	Swelling degree	[39]
	Mechanical properties	[39,41]
CuNPs	Antimicrobial activity In vivo wound healing activity Copper ion release tesr	[42]
ZnO NPs	Antimicrobial activity	[44,47,48,49]
	Cytotoxicity analysis	[49,50]
	In vivo wound healing activity	[45,48,49]
	Swelling degree	[46,49]
	Porosity	[49]
	Mechanical properties	[46,47]
	Water vapor transmission rate	[49]
	Hemocompatibility	[46]
FeO NPs	Antimicrobial activity	[53]
	Porosity	
	Water absorption	
	Iron release	
	Antidiabetic activity	
Fe_3_O_4_ NPs	Antimicrobial activity	[54]
	Cytotoxicity analysis	
	Biocompatibility	
	Swelling degree	
	Porosity	
	Mechanical properties	
	Water vapor transmission rate	
CeO_2_ NPs	Antimicrobial activity	[56]
	Cytotoxicity analysis	
	Evaluation of resistance genes expression in *P. aeruginosa*	
	In vivo wound healing activity	[57]
	Mechanical properties	
	Water absorption	
	In vitro cell proliferation test	
MWCNT_TiO_2_	Antimicrobial activity	[59]
	Biocompatibility	
	Mechanical properties	
CuO NPs	Mechanical properties Antimicrobial activity Biocompatibility	[62]

**Table 4:** The action, the advantages and disadvantages of the nanoparticles used for wound dressing production.

Nanofiller	Effect on Cells	Advantage (+) Disadvantage (−)
AgNPs	Oxidative stress. Superoxide and hydroxyl radical generation	Strong antibacterial action (+) May cause allergies (−) Toxic to human skin keratinocyte cells (−)
AuNPs	Presumably cause oxidative damage to bacteria	Nontoxic to human skin keratinocyte cells (+) Weak antimicrobial effect (−)
CuNPs	Presumably copper ions bind the DNA molecules of a bacterial cell	Nontoxic to human skin keratinocyte cells (+) Lower antimicrobial effect vs. antibiotics (−)
ZnO NPs	Electrostatic attraction of zinc ions to the bacterial cell membrane followed by release of the cell contents	Strong antibacterial action (+) Genotoxic to human epidermal cells (−)
FeO NPs, Fe_3_O_4_ NPs	Penetrate through cell membrane and prevent transmembrane electron trnsfer	Strong antibacterial action (+) Toxic to human skin keratinocyte cells (−)
CeO_2_ NPs	Oxidative stress on lipids and/or proteins in the plasma membrane through reduction in Ce^4+^ to Ce^3+^.	Strong antibacterial action (+) Low toxicity towards human skin keratinocyte cells (+)
TiO_2_ NPs	Oxidative stress. Generation of two reactive oxygen intermediates—OH and H_2_O_2_.	Nontoxic to human skin keratinocyte cells (+) Weak antimicrobial effect (−)
CuO NPs	Oxidative stress. Generation of four reactive oxygen intermediates—the superoxide oxygen radical, ·OH, H_2_O_2_, the singlet oxide.	Strong antibacterial action (+) Toxic to human skin keratinocyte cells (−)

**Table 5:** The application trends for nanoparticles in the preparation of antimicrobial wound dressings.

Nanoparticle	Year	No. of Publications	References
AgNPs	2018	6	[65,66,67,68,69,70]
	2019	4	[71,72,73,74]
	2020	12	[75,76,77,78,79,80,81,82,83,84,85,86]
	2021	2	[23,27]
	2022	13	[22,24,25,26,28,29,30,31,32,33,34,35,36]
AuNPs	2020	2	[87,88]
	2021	2	[39,41]
CuNPs	2020	1	[89]
	2021	1	[42]
ZnO NPs	2018	5	[70,90,91,92,93]
	2019	4	[94,95,96,97]
	2020	2	[85,98]
	2021	2	[44,46]
	2022	5	[45,47,48,49,50]
FeO NPs	2021	1	[53]
Fe_3_O_4_ NPs	2022	1	[54]
CeO_2_ NPs	2019	1	[99]
	2021	1	[56]
	2022	1	[57]
TiO_2_ NPs	2020	1	[100]
	2022	1	[59]
CuO NPs	2021	1	[62]
Cu_2_O NPs	2018	1	[101]
Lignin NPs	2018	1	[102]
Silver zeolite NPs	2018	1	[103]
ZrO_2_ NPs	2020	1	[104]

**Table 6:** The application trends for polymers in the preparation of antimicrobial wound dressings.

Polymer	Year	No. of Publications	References
Chitosan and derivatives thereof	2018	3	[70,93,102]
	2019	4	[71,72,74,94]
	2020	8	[75,76,81,82,86,88,89,100]
	2021	4	[27,42,46,53]
	2022	6	[25,34,35,47,48,49]
Polyvinyl alcohol	2018	4	[65,91,92,102]
	2019	3	[73,94,96]
	2020	5	[81,83,84,87,88]
	2021	3	[39,42,53]
	2022	4	[24,25,26,49]
Cellulose and derivatives thereof	2018	3	[67,69,101]
	2020	2	[85,104]
	2022	2	[33,59]
Polycaprolactone	2020	1	[104]
	2021	3	[41,56,62]
	2022	1	[29]
Gelatin	2020	1	[80]
	2021	2	[56,62]
	2022	3	[30,31,57]
Starch and derivatives thereof	2020	1	[83]
	2022	3	[31,32,49]
Konjac glucomannan	2018	1	[68]
	2020	3	[75,82,85]
Collagen	2020	2	[76,86]
	2022	2	[23,59]
Silk fibroin	2018	2	[91,93]
	2019	1	[71]
Sodium alginate and calcium alginate	2019	1	[97]
	2020	1	[80]
	2022	1	[35]
Hyaluronic acid and derivatives thereof	2020	1	[89]
	2021	1	[27]
	2022	1	[22]
Polyalkylene glycols	2018	1	[69]
	2019	1	[72]
	2020	2	[98,100]
	2022	1	[29]
Keratin	2019	1	[95]
	2020	1	[79]
Polylactic acid	2022	2	[28,57]
κ-carrageenan	2020	2	[85,87]
Polyurethane	2022	2	[28,32]
Oxidized dextran	2021	1	[27]
Polyvinylpyrrolidone	2018	1	[69]
Agar	2018	1	[69]
Poly(acrylic acid-co-itaconic acid)	2018	1	[90]
Nylon 66	2018	1	[66]
Nylon 4/6 copolymer	2018	1	[103]
Galacto-xyloglucan	2020	1	[77]
Gum acacia and carbopol	2020	1	[78]
HBV	2019	1	[99]
Polyacrylonitrile	2022	1	[36]
Heparin	2021	1	[39]
Polyacrylic acid and polyallylamine hydrochloride	2021		[44]
Glycogen	2022	1	[48]
Polyhydroxyethyl methacrylate	2022	1	[54]
Quaternized chitin	2022	1	[28]
